# A Trace Carbon Monoxide Sensor Based on Differential Absorption Spectroscopy Using Mid-Infrared Quantum Cascade Laser

**DOI:** 10.3390/mi9120670

**Published:** 2018-12-18

**Authors:** Chen Chen, Qiang Ren, Heng Piao, Peng Wang, Yanzhang Wang

**Affiliations:** College of Instrumentation & Electrical Engineering, Key Laboratory of Geophysical Exploration Equipment, Ministry of Education of China, Jilin University, Changchun 130026, China; cchen@jlu.edu.cn (C.C.); renqiang15@mails.jlu.edu.cn (Q.R.); piaoheng18@mails.jlu.edu.cn (H.P.); wangpeng18@mails.jlu.edu.cn (P.W.)

**Keywords:** Trace carbon monoxide sensor, mid-infrared spectrum, quantum cascade laser, differential absorption spectroscopy, residual analysis

## Abstract

Carbon monoxide (CO), as a dangerous emission gas, is easy to accumulate in the complex underground environment and poses a serious threat to the safety of miners. In this paper, a sensor using a quantum cascade laser with an excitation wavelength of 4.65 μm as the light source, and a compact multiple reflection cell with a light path length of 12 m is introduced to detect trace CO gas. The sensor adopts the long optical path differential absorption spectroscopy technique (LOP-DAST) and obtains minimum detection limit (MDL) of 108 ppbv by comparing the residual difference between the measured spectrum and the Voigt theoretical spectrum. As a comparison, the MDL of the proposed sensor was also estimated by Allan deviation; the minimum value of 61 ppbv is achieved while integration time is 40 s. The stability of the sensor can reach 2.1 × 10^−3^ during the 2 h experimental test and stability of 1.7 × 10^−2^ can still be achieved in a longer 12 h experimental test.

## 1. Introduction

Compared to traditional bipolar semiconductor lasers, quantum cascade lasers (QCLs) offer unique advantages of good monochromaticity, high quantum efficiency, good temperature stability, flexible wavelength design, and fast response [1,2,3]. In addition, the infrared spectrum of QCL covers three important atmospheric transmission windows. Therefore, QCLs have an incomparable advantage over other luminous sources in the field of gas detection [4,5,6].

In recent years, the application of QCL to detect trace gas in the infrared "fingerprint region" has been developing rapidly. In 2008, J. B. Mcmanus et al. at the Aerodyne research center, adopted a QCL with center wave number of 967 cm^−1^ combined with a long optical path direct absorption method to detect ammonia gas. The light path length of multiple reflecting cell was increased to 76 m, and the minimum detection limit (MDL) of ammonia gas was achieved as 0.2 ppbv [7]. In 2013, P. G. Carbajo et al. detected H_2_CO using a QCL with central wave number of 1769 cm^−1^. The MDL was improved to 0.06 ppbv by using the optical feedback-cavity enhanced absorption spectrum technology [8]. In 2010, K. Ruifeng et al. utilized the pulsed QCL with the central wave number of 1904 cm^−1^ combined with direct absorption spectrum detection technology to detect nitric oxide gas [9]. The MDL of nitric oxide reached 3.4 ppmv by performing the least squares calculation method to fit baseline. In 2014, L. Guolin et al. reported a CO_2_ sensor using the 4.8 μm mid-infrared QCL to realize a MDL of 180 ppbv [10]. 

The optimization work on open gas cell using ellipsoid condensers is the main contribution here. Although the mentioned sensors achieved good detection performance for target gases, the shortcoming of complicated structure limits their usage in field applications. In this paper, a new CO sensor using mid-infrared QCL with center wavelength of 4.65 μm is introduced. The sensor utilizes a 12 m long optical path differential absorption spectroscopy technique (LOP-DAST) to achieve MDL of 108 ppbv, minimum Allan deviation of 61 ppbv, and high performance of working stability during the long experimental test. To resolve the issue of large packages of core constituted components, chip-level mid-infrared gas sensors with high sensitivity is discussed in the final section, which appears very promising for future gas sensing.

## 2. Detection Principle of CO Using LOP-DAST

### 2.1. Selection of CO Absorption Line

**Figure 1:**
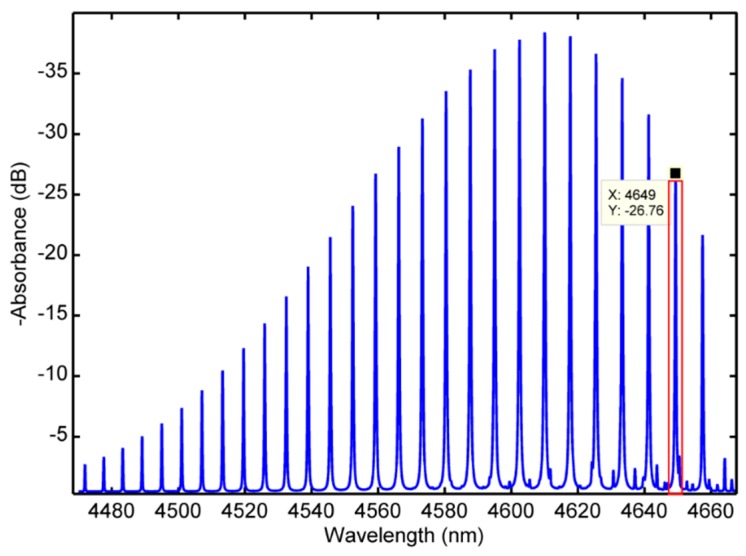
Absorption spectrum of CO.

In the mid-infrared spectrum band, the absorption spectrum of gas molecules generally includes rotational spectrum and rotational vibration spectrum, and each gas has many absorption bands in the mid-infrared spectrum band [11,12,13]. Taking CO as a target, the absorption spectrum band around 4.6 μm caused by the fundamental frequency vibration of CO molecules is shown in Figure 1.

As shown in Figure 1, the x coordinate is wavelength (nanometers, nm), the y coordinate is absorption intensity (decibel, dB), and blue lines represent CO gas absorption lines. Due to the limitation of tuning ability of mid-infrared QCL adopted in this paper, the optimum luminous wavelength is chosen at 4.65 μm, which is close to the peak value of CO absorption spectrum at 4.649 μm, as illustrated in the red box. For this absorption spectrum line, the absorption coefficient of CO is −26 dB and is in the atmospheric window range. In addition, the improved accuracy of measured results can be reached because of spectral interference exclusion from other atmospheric components, such as water, methane, and CO_2_.

### 2.2. Derivation of LOP-DAST technique

The LOP-DAST can be described as follows. According to the Beer-Lambert law, absorbance is proportional to the concentrations of the attenuating species in the material sample [14,15,16,17,18]. The light intensity after passing through the gas absorption cell with light path length *L* is:

(1) $$I\left(\lambda \right)=I_{0}\left(\lambda \right)exp\left(\phi \left(\sigma_{i},C_{i},L,\varepsilon_{r},\varepsilon_{m}\right)\right)$$

The multiple variables function in the above equation can be expressed as:

(2) $$\phi \left(\sigma_{i},C_{i},L,\varepsilon_{r},\varepsilon_{m}\right)=\sum_{i=1}^{n}\sigma_{i}\left(\lambda \right)C_{i}L+\varepsilon_{r}\left(\lambda \right)+\varepsilon_{m}\left(\lambda \right)$$

Here, *λ* is the wavelength of luminous light, *σ_i_*(*λ*) denotes the absorption cross section of the *i*th gas, *C_i_* is the average concentration of the *i*th gas, *L* is the light path length, *ε_r_*(*λ*) is Rayleigh scattering, *ε_m_*(*λ*) is Mie scattering. The absorption cross section of Rayleigh scattering and Mie scattering are shown as a broadband absorption cross section. The LOP-DAST method decomposes the absorption cross section into broadband absorption cross section and narrow absorption cross section, namely:

(3) $$\sigma_{i}\left(\lambda \right)=\sigma_{i}^{b}\left(\lambda \right)+\sigma_{i}^{\prime }\left(\lambda \right)$$

where, $\sigma_{i}^{b}\left(\lambda \right)$ represents the broadband absorption cross section of the *i*th gas, and $\sigma_{i}^{\prime }\left(\lambda \right)$ represents the narrow absorption cross section of the *i*th gas. Therefore, Formula (2) can be divided into the following form:

(4) $$\phi^{b}\left(\sigma_{i}^{b},C_{i},L,\varepsilon_{r},\varepsilon_{m}\right)=\sum_{i=1}^{n}\sigma_{i}^{b}\left(\lambda \right)C_{i}L+\varepsilon_{r}\left(\lambda \right)+\varepsilon_{m}\left(\lambda \right)$$

(5) $$\phi^{\prime }\left(\sigma_{i}^{\prime },C_{i},L\right)=\sum_{i=1}^{n}\sigma_{i}^{\prime }\left(\lambda \right)C_{i}L$$

By means of numerical filtering, the terms *ϕ^b^* only containing broadband absorption cross section can be removed, so as to obtain the differential optical density of the measured gas,

(6) $${\left(O.D.\right)}_{\lambda }=ln\left[\frac{I\left(\lambda \right)}{I_{0}\left(\lambda \right)}\right]=\sum \sigma_{i}^{\prime }\left(\lambda \right)C_{i}L$$

The CO concentration in absorption cell can be obtained by fitting the differential optical density with the reference spectrum via the least square method.

The above analysis shows that obtaining the reference absorption cross section in the absorption spectrum band is key to measuring the CO concentration. The CO absorption line shape at ambient temperature and a bar pressure given by HITRAN database are used to calculate the Voigt theoretical spectrum. The Voigt theoretical spectrum takes into account the Lorentz lineshape generated by gas collision, spontaneous radiation and Doppler lineshape generated by velocity distribution of luminous particles. The comprehensive function is as follows:

(7) $$\begin{array}{cc} g_{z}\left(\nu ,\nu_{0}\right) & =\int_{-\infty }^{+\infty }g_{L}\left(\nu_{1},\nu_{0}\right)g_{D}\left(\nu ,\nu_{1}\right)d\nu_{1}\\ & =\frac{1}{\pi }\sqrt{\frac{ln2}{\pi }}\int_{-\infty }^{+\infty }\frac{\alpha_{L}}{{\left(\nu_{1}-\nu_{0}\right)}^{2}-\alpha_{L}^{2}}\frac{1}{\alpha_{D}}exp\left(-\omega \left(\nu_{1}\right)\right)d\nu_{1} \end{array}$$

where,

(8) $$\omega \left(\nu_{1}\right)=\frac{ln2}{\alpha_{D}^{2}}{\left(\nu -\nu_{1}\right)}^{2}$$

$\alpha_{L}$ and $\alpha_{D}$ are the half-width of Lorentz lineshape and Doppler lineshape, respectively. $\nu_{0}$ is the central frequency. If $\alpha_{D}$ is set as a constant [19], then:

(9) $$g_{z}\left(\nu ,\nu_{0}\right)=\sqrt{\frac{ln2}{\pi }}\frac{1}{\alpha_{D}}\left(\frac{\mu }{\pi }\int_{-\infty }^{+\infty }\frac{1}{{\left(\xi -t\right)}^{2}-\mu^{2}}exp\left(-t^{2}\right)dt\right)$$

where,

(10) $$\xi =\sqrt{ln2}\frac{\nu -\nu_{0}}{\alpha_{D}},\;\mu =\sqrt{ln2}\frac{\alpha_{L}}{\alpha_{D}},\;t=\sqrt{\frac{ln2}{\alpha_{D}}}\left(\nu_{1}-\nu \right)$$

The Voigt absorption spectrum has been widely used in differential absorption spectrometry. The MDL of the sensor can be determined by comparing the residual between the spectrum measured in the experiment and the Voigt theoretical spectrum.

## 3. Sensor Configuration

**Figure 2:**
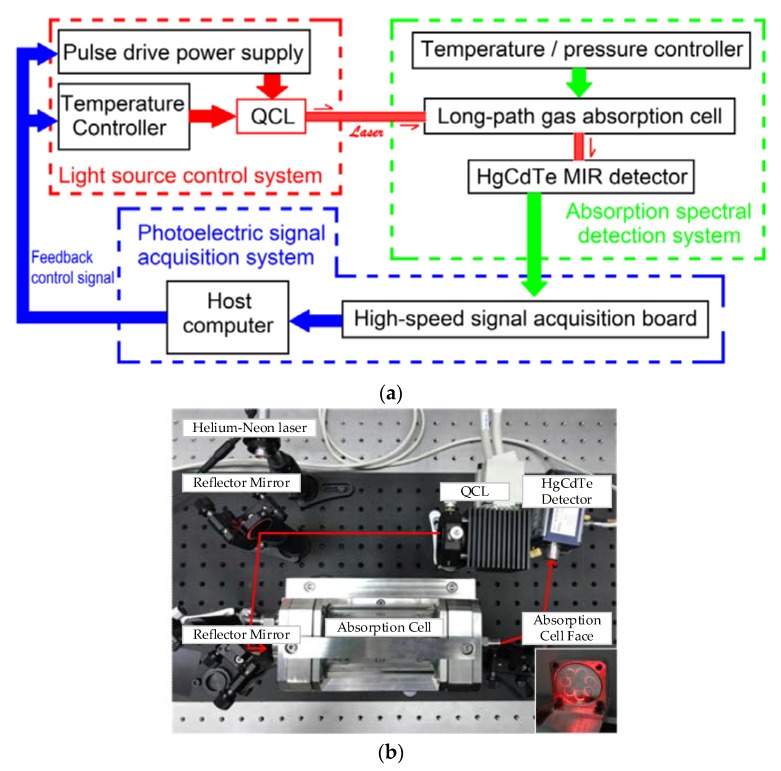
(**a**) The block diagram of CO sensor. (**b**) Physical image of CO sensor.

The core device of a gas sensor is a QCL with a center wavelength of 4.65 μm. The luminous mid-infrared light is focused on the 12 m long optical path absorption cell (made of stainless steel) through the two reflected-gilt spherical lens. Then, the mid-infrared light emitted from the absorption cell passes through a paraboloid reflector and is received by a liquid nitrogen cooled HgCdTe detector; the response peak wavelength is 5 μm, the spectral response range is from 2 μm to 12 μm, the response time is less than 100 ns, and the size of the detection surface is 1 mm^2^. The overall design block diagram and physical image of the CO sensor is shown in Figure 2a,b, respectively.

In the control system, the self-designed constant power controller for QCL is utilized, which is capable of the adjustment of width and duty ratio of the output driving pulse. The peak driving current is 10 A, and the pulse rising/falling time is less than 10 ns. The laser is real-time controlled in a constant power model by reading the feedback current from the photodiode packaged in the inner QCL. The QCL working temperature fluctuation is less than ±0.05 °C by using adaptive proportional integral derivative (PID) algorithm, and no overshoot control of target working temperature is realized.

The optical part is composed of a gold-plated reflector, multiple reflection mirror, and a mid-infrared HgCdTe detector. The reflector selects platinum materials as the coating, which have a higher than 99% reflectivity for mid-infrared light. The light path length of the absorption cell used in the sensor is 12 m, the volume is 500 mL, the operating reflection band range is 2.5 μm to 10 μm, and the physical size is 450 mm × 110 mm × 110 mm. Due to the invisibility of mid-infrared, the luminous light emitted from QCL is collimated via a visible red light from the Helium-Neon laser. As a result, most of the mid-infrared light can be reflected through an all-reflecting spherical lens into the inlet aperture of the absorption cell. Finally, optical output signals can be detected by the HgCdTe detector after several passes in the absorption cell.

## 4. Experiment

Under the conditions of ambient temperature (293 K) and one bar pressure (1.0 × 10^5^ Pa), the performance indicators of trace CO sensor, the concentration-voltage response, measurement MDL, and working stability are tested. MDL performance is assessed by comparing the residual difference method and the Allan deviation method.

### 4.1. Response

**Figure 3:**
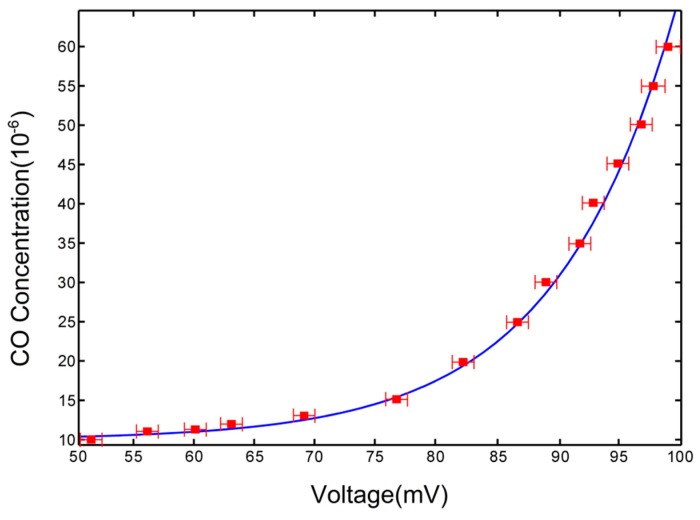
Concentration of CO versus output voltage of the sensor.

In order to observe the response of the proposed sensor, 15 CO gas samples with different standard concentrations (10 ppmv to 60 ppmv) were prepared by dynamic gas dilution equipment. The prepared CO gases are pumped into the gas cell successively, and the relation between output voltage signal of the sensor and CO concentration was obtained, as shown in Figure 3.

In the figure, the x coordinate represents the output voltage collected by the photoelectric signal acquisition system, the y coordinate describes the concentration of the CO gas sample, and the solid blue line is the fitting curve. It can be seen from the figure that the measured gas concentration with respect to the output voltage is exponential, which is in line with Bill lambert’s law, as shown in Formula (1).

### 4.2. Stability 

**Figure 4:**
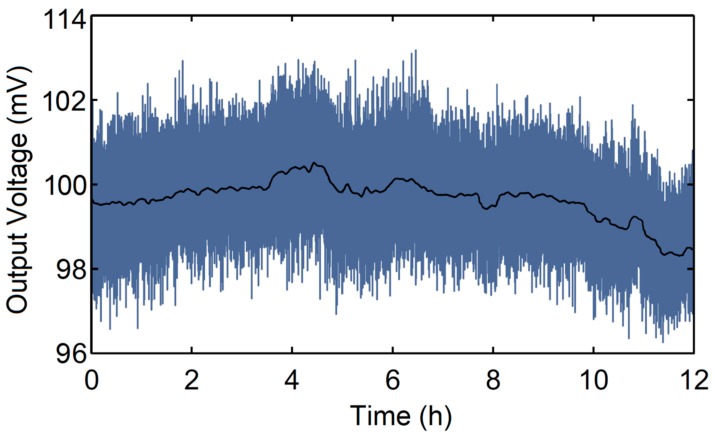
The measured data of 60 ppmv CO over 12 h.

Stability performance can be obtained by measuring the sensor’s output changes along with time and a certain concentration of measured gas in absorption cell. The CO gas with a concentration of 60 ppmv was observed for 12 h, and the relationship between the output voltage corresponding to CO gas concentration and the detection time was recorded; the results are shown in Figure 4.

The blue curve is the output voltage collected per second, and the black curve is the average value of the collected data with integral time of 30 s. It can be seen from the figure that the sensor had stable operations within two hours of starting up, the recorded data fluctuated with 0.4 mV, and stability was as high as 2.1 × 10^−3^. Accordingly, the response amplitude of the sensor changed within 1.6 mv in 12 h, and the stability of 1.7 × 10^−2^ was achieved in the longer experimental test. In future work, reference cells can adapt to suppress common noise, which leads to stability promotion of the proposed sensor.

### 4.3. Minimum Detection Limit

**Figure 5:**
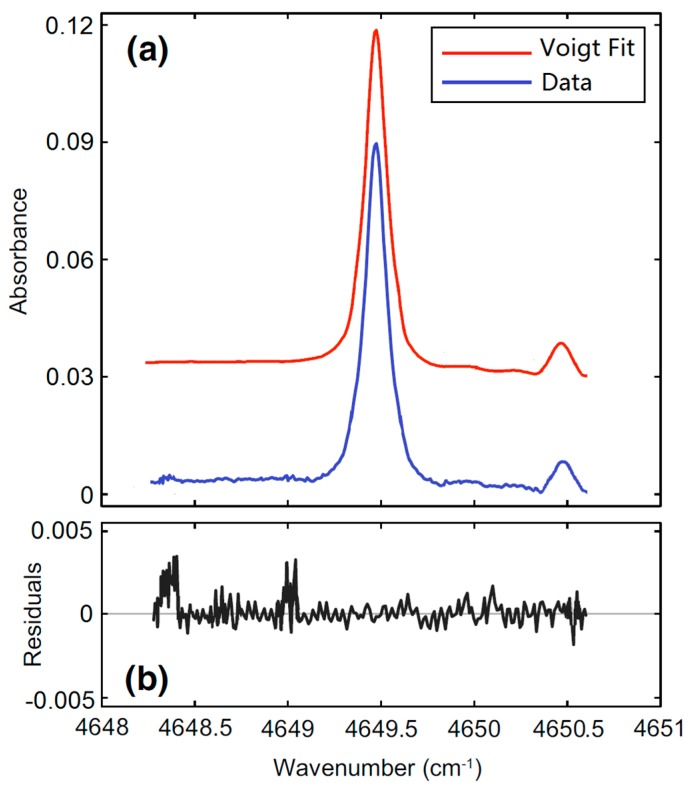
(**a**) Measured absorption spectrum (blue line) and Voigt theoretical spectrum (red line) via the LOP-DAST method. (**b**) Residual curve calculated by aforementioned two spectrums.

MDL is defined as the gas concentration when the effective gas absorption signal is distinguishable from background noise, namely when Signal-Noise-Ratio (SNR) is 1. MDL of the proposed sensor was estimated by comparing the residual difference between the measured spectrum and the Voigt theoretical spectrum. The experimental results to measure 10 ppmv CO is shown in Figure 5.

The red curve in Figure 5a is the Voigt theoretical spectrum; the difference curve between the measured absorption spectrum and the base line background is shown below (blue line). The coincidence degree between the Voigt theoretical spectrum and the difference curve is very good, the residual error less than ±0.5%. We investigated the noise spectrum near the absorption peak and found that the noise standard error (SE) is 9.395 × 10^−4^. Here, we can approximately assume that this SE value is the minimum detectable concentration when the Signal-Noise-Ratio (SNR) equals to 1. In addition, the magnitude of absorption spectrum peak is 0.087, so the SNR = 0.087/SE = 92.6. Due to the measured CO concentration being 10 ppmv, the MDL of the sensor can be calculated as 10 ppmv/92.6 = 108 ppbv.

Due to the characteristics of non-stationary and slow-time variation when the sensor worked, we introduced Allan deviation to estimate MDL more accurately. Allan deviation has been widely used in the estimation of MDL of gas sensor [20,21], and its expression is:

(11) $$\sigma_{A}^{2}\left(\tau \right)=\frac{1}{2\left(N-2\right)\tau^{2}}\sum_{i=1}^{N-2}{\left(X_{i+2}-2X_{i+1}+X_{i}\right)}^{2}$$

**Figure 6:**
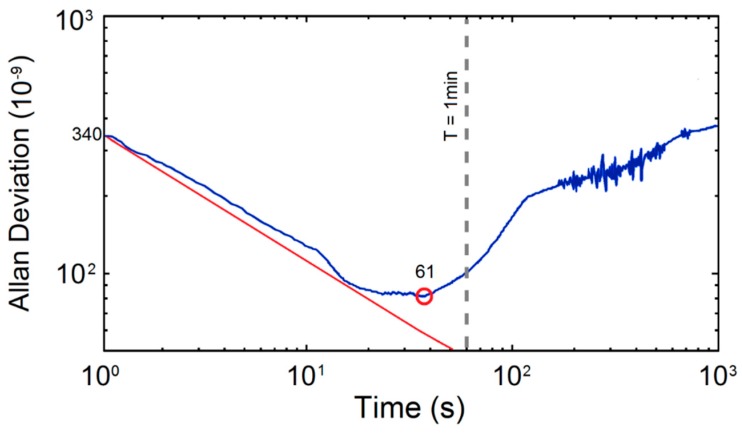
Allan deviation of the acquisition data of 60 ppmv CO.

*N* is the sampling sequence of gas concentration in time domain, τ is the sampling period, *X_i_* is the sampling value. We measured the CO gas samples of 60 ppmv and calculated the Allan deviation of the acquisition data; the results are shown in Figure 6.

It can be seen from the figure that the initial value of Allan deviation was about 340 ppbv, and the minimum value reached 61 ppbv with integration time of 40 s. This result is also similar to the value obtained from previous residual analyses.

## 5. Discussion and Conclusions

In this paper, a high-performance trace CO sensor using mid-infrared QCL combined with LOP-DAST is introduced. The working principle of LOP-DAST and calculating method of the absorption cross section are described, respectively. The stability of the sensor reached 1.7 × 10^−2^ by testing standard gas sample for a long period, and the MDL achieved approximate 100 ppbv by comparing the residual difference method and the Allan deviation method. Besides, the sensor is capable of detecting a variety of trace gases by changing the luminous source with different excitation wavelengths.

Comparing to state-of-the-art trace gas sensors using mid-infrared luminous source, simplified optical structure is utilized to achieve miniaturization and practicality, and maintain high measurement detection performance. Nevertheless, the shortcoming of the bulky package of the optical core devices and the auxiliary modules is obvious, such as bulky electrical power supply and Peltier temperature controller for QCL and HgCdTe detector, and multi-reflection cell with the size similar to a shoe box. Although the described sensor obtained ppbv levels of MDL, it was too large to be convenient for some special applications with compact volume requirement, such as gas detection in mobile and airborne platforms. 

To meet the compact requirements of these specific applications, the challenge of fabrication technique on a chip level is of critical importance. Fortunately, the core components of the described mid-infrared gas sensor have been minimized, such as mid-infrared MEMS luminous source [22], micro-cavity absorption cell using silicon microring resonators [23,24], and on-chip HgCdTe photodiode detectors [25]. The advanced comments combined with integrated packaging technology to constitute new concept sensors on a chip level can potentially rival current infrared absorption spectrum sensors. Thus, micro mid-infrared gas sensors with ultra-compact size and high sensitivity appear to be very promising for future gas sensing.
